# Indomethacin: Effect of Diffusionless Crystal Growth on Thermal Stability during Long-Term Storage

**DOI:** 10.3390/molecules28041568

**Published:** 2023-02-06

**Authors:** Roman Svoboda, Nicola Koutná, Daniela Košťálová, Miloš Krbal, Alena Komersová

**Affiliations:** 1 Department of Physical Chemistry, Faculty of Chemical Technology, University of Pardubice, Studentská 573, 532 10 Pardubice, Czech Republic; 2 Center of Materials and Nanotechnologies (CEMNAT), Faculty of Chemical Technology, University of Pardubice, nam. Cs legii 565, 530 02 Pardubice, Czech Republic

**Keywords:** amorphous indomethacin, crystallization, kinetic prediction, particle size, storage

## Abstract

Differential scanning calorimetry and Raman spectroscopy were used to study the nonisothermal and isothermal crystallization behavior of amorphous indomethacin powders (with particle sizes ranging from 50 to 1000 µm) and their dependence on long-term storage conditions, either 0–100 days stored freely at laboratory ambient temperatures and humidity or placed in a desiccator at 10 °C. Whereas the γ-form polymorph always dominated, the accelerated formation of the α-form was observed in situations of heightened mobility (higher temperature and heating rate), increased amounts of mechanically induced defects, and prolonged free-surface nucleation. A complex crystallization behavior with two separated crystal growth modes (originating from either the mechanical defects or the free surface) was identified both isothermally and nonisothermally. The diffusionless glass–crystal (GC) crystal growth was found to proceed during the long-term storage at 10 °C and zero humidity, at the rate of ~100 µm of the γ-form surface crystalline layer being formed in 100 days. Storage at the laboratory temperature (still below the glass transition temperature) and humidity led only to a negligible/nondetectable GC growth for the fine indomethacin powders (particle size below ~150 µm), indicating a marked suppression of GC growth by the high density of mechanical defects under these conditions. The freely stored bulk material with no mechanical damage and a smooth surface exhibited zero traces of GC growth (as confirmed by microscopy) after >150 days of storage. The accuracy of the kinetic predictions of the indomethacin crystallization behavior was rather poor due to the combined influences of the mechanical defects, competing nucleation, and crystal growth processes of the two polymorphic phases as well as the GC growth complex dependence on the storage conditions within the vicinity of the glass transition temperature. Performing paired isothermal and nonisothermal kinetic measurements is thus highly recommended in macroscopic crystallization studies of drugs with similarly complicated crystal growth behaviors.

## 1. Introduction

Amorphous active pharmaceutical ingredients (APIs) are among the top sought-out solutions for increasing the bioavailability of modern drugs [1,2,3,4,5]. The disordered state of amorphous/glassy materials significantly (often multifold) enhances the dissolution of the API in the bloodstream, being particularly beneficial in the case of drugs of low solubility [6,7]. However, amorphous APIs are also associated with a significant disadvantage in the instability of the glassy state. Under certain conditions (e.g., increased temperature or humidity during long-term storage), these amorphous materials can “spontaneously” crystallize, which can largely decrease their availability in the patient’s body and cause substantial harm (via prolonged release and/or decreased total received dose of the API) [8,9,10,11]. This makes the thermal stability and crystal growth behavior of amorphous APIs the key factors in their practical utilization.

Indomethacin (IMC) belongs to the arylalkanoic family derived from the 2-arylacetic acids. Due to its anti-inflammatory effects and nonsteroidal nature, IMC is commonly used to treat inflammation, chronic rheumatoid arthritis, periarthritis, osteoarthritis, spondylosis deformans, and acute gout (the pharmaceutical mechanism is based on the inhibition of cyclooxygenase, an enzyme from the prostaglandin synthetic cascade) [12,13,14]. The clinical effectiveness of IMC is, however, somewhat hindered by its low water solubility (0.937 mg·L^−1^ at 25 °C [15]), which makes it an ideal candidate for an amorphization-based route of availability improvement. Since IMC is commonly used as a water-insoluble model API, it is surprising that its crystallization behavior has not yet been fully explored. Several published studies deal in detail with the microscopic [16,17,18] as well as macroscopic [19,20,21] crystal growth characterization of IMC, but neither of these papers addresses the key question of the influence of particle size and mechanically induced defects on the crystallization tendency of amorphous IMC, which is crucial for the determination of its long-term stability during storage and the identification of its safe processing conditions. In addition, neither of the mentioned papers deal with the conditions for long-term storage, which are also essential for the stability of the amorphous phase. In this regard, only papers represented, e.g., by [22], can be found partially dealing with the amorphization of the crystalline IMC by milling—in [22], the kinetics of surface and volume amorphization of crystalline IMC were described by the first-order model, and milling for 120 min was sufficient to obtain fully amorphous powdered IMC material. The relationship between the particle size of the powdered amorphous APIs and their tendency toward crystallization is crucial for appropriate treatment during the processing and long-term storage of these materials. It has been recently shown in the case of Enzalutamide [23,24] that the kinetic analysis of the nonisothermal crystallization data can be very useful in accurately predicting crystal growth under extrapolated conditions. Consequently, we wanted to explore the power of the state-of-the-art kinetic analysis methods for the significantly more difficult case of IMC, which exhibits the simultaneous formation of several polymorphic forms and has a low glass transition temperature T_g_, enabling a diffusionless sub-T_g_ crystal growth [20] during the processing and storage of the material.

This paper is a sequel to the previous paper, which included more theoretical work on the general thermokinetic characterization of IMC [25], described all thermally induced phenomena observable in IMC, and introduced the hypotheses about their mutual relationships. This paper focuses on practical questions associated with the processing, and short- and long-term storage of various forms of amorphous IMC. In this paper, the influence of particle size on the thermal stability and crystallization behavior of powdered amorphous IMC (prepared by the melt-quenching procedure) will be described based on the isothermal and nonisothermal calorimetric data. The main aim of the included research is to explore the influence of long-term storage of IMC on its crystallization kinetics under different storage conditions. Apart from the advanced kinetic analysis of the crystallization data, the correspondence between the kinetics determined for the high-temperature nonisothermal crystal growth and the kinetics of the short-term and long-term isothermal annealing (storage) will be explored. Here, one of the main aims will be to test the current state-of-the-art methods of kinetic analysis and their predictive capabilities in this relatively difficult scenario (as described in the previous paragraph). Particular attention will also be paid to the practical consequences of storage under various conditions on the crystallization behavior of amorphous IMC.

## 2. Experimental

Amorphous indomethacin was prepared using the melt-quenching routine [25] from 5N crystalline material (Sigma-Aldrich). Amorphous product was powdered into the following fractions: 20–50, 50–125, 125–180, 180–250, 250–300, and 300–500 µm. Larger bulk pieces (with the largest dimension ~500–1000 μm) were denoted as “bulk”; their average dimension was set to d_aver_ = 1000 μm. The powdering of amorphous IMC was performed either by gentle tapping, which caused disintegration due to the internal tensions from the quench, or it was heavily ground with an agate pestle and mortar—these two batches of IMC powders will be further denoted as tIMC (tapped), and gIMC (ground). The powders were sieved (Retsch sieves) with no added pressure.

The prepared amorphous IMC powders were stored under two types of conditions: in a desiccator at 10 °C; and as a freely stored powder at laboratory temperature and humidity conditions. The effect of long-term storage was explored by performing a series of characterization measurements at three-time intervals: immediately after the preparation of the IMC bulk ingot, after 14 days, and after 100 days from preparation. The calorimetric crystallization data were obtained through differential scanning calorimetry (DSC), performed using a heat flow DSC instrument (Q2000, TA Instruments, New Castle, DE, USA). [25] The DSC heating scans were performed in the 20–180 °C range at heating rates (q^+^) of 0.5, 1, 2, 5, 10, and 20 °C·min^−1^. Sample masses were approximately 2 mg (accurately weighted to 0.01 mg); hermetically sealed low-mass T-zero DSC pans were used. Isothermal annealing was performed at temperatures T_a_ = 70, 75, 80, 85, 90, and 95 °C, where the samples were heated from 25 °C to T_a_ at 50 °C·min^−1^. Selected DSC measurements were repeated to confirm the reproducibility of the data.

The IMC powders were further characterized using a micro X-ray diffraction device, XRD (Empyrean Malvern Panalytical, Malvern, United Kingdom), in the 5–40° range, an optical microscope iScope PLMi (Euromex, Arnhem, The Netherlands) in reflection mode (equipped with ×40 and ×80 high-quality objectives and a Moticam visual camera), a thermogravimetric instrument STA (TGA) 449 F5 Jupiter (Netzsch, Selb, Germany) equipped with DSC/TG holder, and a DXR2 Raman microscope (Nicolet, ThermoFisher Scientific, Prague, Czech Republic) equipped with a 785 nm excitation diode laser (laser spot size of 1.6 μm) and CCD detector (5 mW laser power, 3 s scan duration, 100 scans per spectrum).

## 3. Results

This section is divided according to the experimental techniques used for the IMC powder characterizations.

### 3.1. Differential Scanning Calorimetry

**Figure 1:**
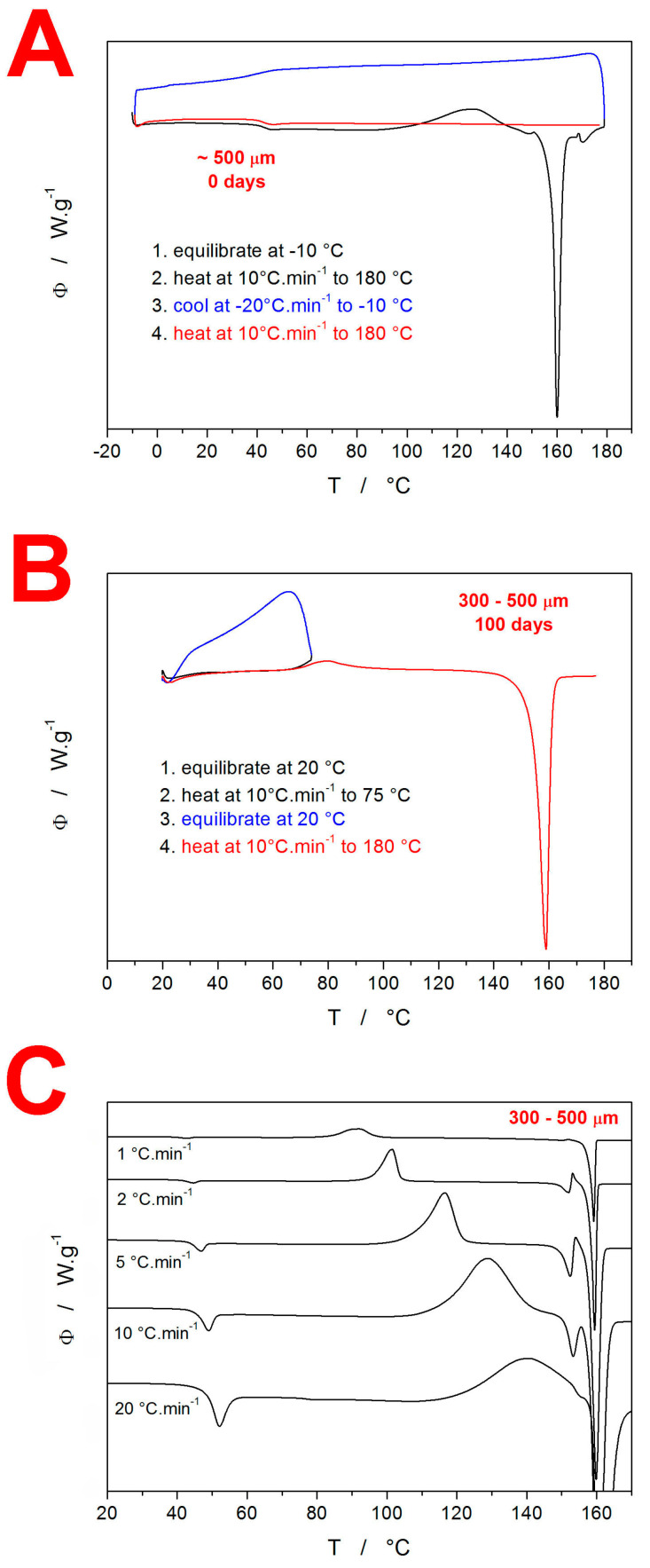
(**A**) Glass-forming classification experiment performed using DSC for amorphous IMC. Exothermic signals evolve in the upwards direction. (**B**) Partial crystallinity acceleration test performed for amorphous IMC. (**C**) Example of the series of kinetic measurements performed as simple DSC heating scans of the amorphous IMC powder at different q^+^.

Differential scanning calorimetry was used to investigate the crystallization behavior of amorphous IMC. In the first series of measurements, the basic features of the IMC crystallization behaviors were verified—see the DSC curves in Figure 1. In Figure 1A, the classification experiment for the drug glass-forming ability [26,27] is demonstrated. This experiment consisted of three steps: (1) heating at 10 °C·min^−1^ above the melting temperature T_m_; (2) cooling at 20 °C·min^−1^ below the glass transition temperature T_g_; and (3) heating at 10 °C·min^−1^ above the melting temperature T_m_. If the material, as in this case, did not crystallize after the re-melting either during the cooling or during the consequent heating step, it was categorized as class III GFA material. Note that during the cooling period after the melt, the absence of exothermic signals indicates no crystal growth on the potentially formed nuclei, and the undercooled melt freezes during the glass transition. During consequent heating, the glassy material softens and loosens above T_g_, and again, no exothermic signals occur. Further heating then leads to a continuous decrease in the material’s viscosity, and the structure gradually changes from the undercooled melt to a melt in the T_m_ region. The absence of the formation of a crystalline phase also means that no melting peak occurs. The second basic feature of the IMC crystallization behavior is depicted in Figure 1B. This experiment demonstrated the simplicity and continuity of the crystal growth in amorphous IMC, where the presence of a crystalline phase within the semicrystalline matter (as formed during the preceding heating to 75 °C and immediate cooling back below T_g_) does not influence/catalyze the consequent thermally initiated crystal growth during the repeated heating. This behavior is verified by the similarity of the onsets of the crystallization peak during the first and second heating steps. The third basic feature of the IMC crystallization behavior is shown in Figure 1C. This graph demonstrates the kinetics of the base thermokinetic phenomena initiated by the heating of the amorphous phase; the 300–500 µm powder fraction heated at different q^+^ was chosen as an example. The endothermic glass transition signal at 45–50 °C only slightly shifts with increasing q^+^, which indicates a high activation energy for this process—in [25], this value was found to be 342 ± 7 kJ·mol^−1^. The second effect on the DSC curves is exothermic and corresponds to the macroscopic manifestation of crystal growth. The crystallization peak not only shifts quite significantly with q^+^ (which indicates significantly lower activation energy than exhibited by the glass transition) but also changes its asymmetry from negatively skewed to positively skewed, which indicates a change in the crystallization mechanism. Moreover, the shift of the crystallization peak with q^+^ is so large that at 20 °C·min^−1^, the material does not manage to turn fully crystalline before T_m_ is reached, and the two processes interfere. The melting peak also apparently changes with the applied q^+^, but only as a consequence of different crystalline phases/polymorphs being formed within the crystallization process. As shown in Figure 1C, the melting of the IMC can be described by two melting peaks—a pre-peak at ~149 °C and the main peak at ~157 °C. Note that the melting pre-peak can be followed by recrystallization (from the melt) as the melted metastable polymorph changes (crystallizes into) the thermodynamically stable polymorph. The two melting peaks correspond to the α-IMC polymorph (149 °C) and γ-IMC polymorph (157 °C) [28,29].

**Figure 2:**
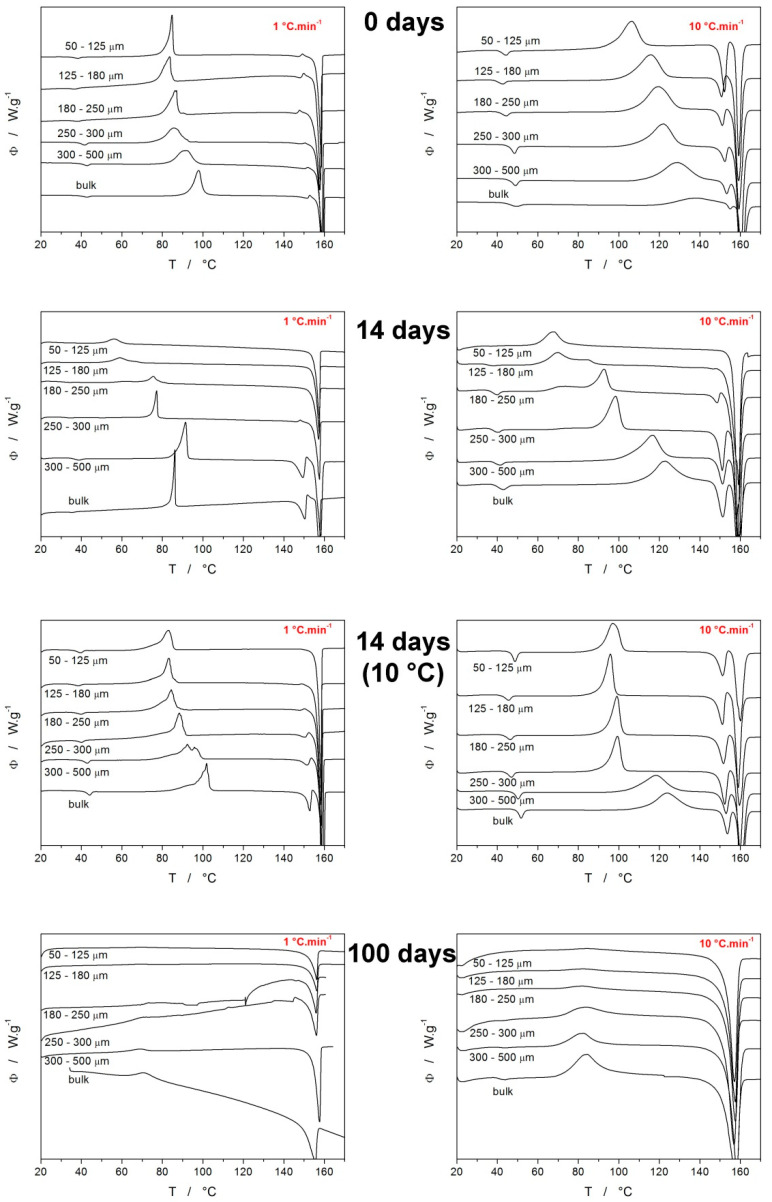
Example DSC curves obtained at 1 and 10 °C·min^−1^ for various IMC powders with defined particle sizes. Each row of graphs corresponds to particular time and temperature storage conditions (powders were stored at laboratory temperature unless otherwise stated). Exothermic signals evolve in the upwards direction.

The main goal of this paper was to explore the crystallization behavior of IMC with respect to the particle size and aging (long-term storage) conditions—the majority of the corresponding DSC data are summarized in Figure 2. However, before interpreting the DSC data from Figure 2, some basic facts [25,28,29] about the IMC polymorphic behavior need to be introduced: Nucleation below 55 °C dominantly produces the γ-IMC form, whereas, above 55 °C, the α-IMC nucleation is preferential. The crystal growth rate of the α-IMC form starts to exceed that of the γ-IMC form above 50 °C; the difference increases with rising T. The metastable α-IMC polymorph has lower interfacial energy σ (compared to the γ-IMC) [30], which results in preferential formation of the α-IMC nuclei in the cases of high molecular mobility [28,31].

As a result, thermodynamically stable IMC polymorphs tend to form during slow crystallization, and the metastable polymorphs are produced during rapid amorphous-to-crystalline transformations. In addition, IMC is known to exhibit a so-called diffusionless glass–crystal GC growth, which manifests itself below T_g_. It is also noteworthy that crystal growth in IMC initiates at cracks, microcracks, and other mechanically induced defects—pure IMC bulk with a smooth surface is extremely stable (as also evidenced by Figure 1A) [18,20,32,33,34,35].

Each graph in Figure 2 displays example curves obtained for the given q^+^ (either 1 or 10 °C·min^−1^) and the time/type of storage for all sizes of prepared amorphous IMC powders. The two graphs obtained for the as-prepared IMC (0 days) show that at low q^+^, the onset of the crystallization process occurs at ~75 °C for the majority of the powders; only the two most coarse powders exhibited a slower crystallization rate, and its onset shifted to a higher T due to the lower amount of available crystallization centers (internal cracks and surface defects). The uniformity of the onsets observed for the fine powders indicates that the effect of the crystallization accelerated by the presence of mechanical defects is saturated at relatively low amounts of these defects. Taking into account the nucleation and growth proceeding primarily at low T during the slow heating at 1 °C·min^−1^, it is understandable that the ratio of the two melting peaks results in absolute dominance of the γ-IMC polymorph formation. On the other hand, at 10 °C·min^−1^ heating, the time that the material spends nucleating at T < 55 °C is very limited, and also, the growth shifts to T ∈ <95 °C; 145 °C>. This results in a significantly higher portion of the α-IMC phase being formed, as evidenced by the considerably larger melting pre-peak (as compared to the main melting peak). It is also noteworthy that the α/γ ratio is highest for the finest powders, which indicates that the presence of defects favors the formation of the faster-growing crystallites (the growth rate is higher for the α polymorph above 50 °C [36]).

The next set of graphs in Figure 2 (denoted “14 days”) correspond to the DSC crystallization data for the samples that were left at laboratory temperature (~25 °C) and humidity for 14 days. The storage has the following consequences: The two finest powders (50–125 and 125–180 µm) crystallize at significantly lower temperatures ~50–60 °C solely into the γ-IMC polymorph, which indicates that the storage-associated nucleation below T_g_ is largely accelerated by the presence of mechanical defects. With the increasing particle size of the powders, it is the formation of the metastable α-IMC phase that gets accelerated compared to the measurements for the as-prepared IMC. This may suggest that although the γ-IMC sub-T_g_ nucleation is dominant, over time, a significant number of α-IMC nuclei develop at the free surface or locations with a low density of defects and thus higher molecular mobility. 

The growth of the metastable α-IMC phase from these nuclei is then sustained by the higher temperatures reached by a faster q^+^. Regarding the humidity, it should theoretically favor the formation of the metastable α-IMC phase, which should, in such a case, increasingly occur at a low d_aver_, where the surface/volume ratio and water vapor adsorption are greatest. The opposite was true; hence we assume that the temperature and mechanical defects play a much greater role in the nucleation process in amorphous IMC. The negligible effect of humidity and its potential adsorption onto the powdered IMC was also tested through thermogravimetric measurements, which showed no mass loss in the 25–100 °C temperature range for all long-term-stored IMC samples—see Figure S1 in the Supplemental Materials for details.

The third set of graphs in Figure 2 (denoted “14 days, 10 °C”) correspond to the DSC crystallization data for the samples that were stored in a desiccator at 10 °C for 14 days. For low q^+^, the positions of the crystallization peaks are much more reminiscent of those obtained for the as-prepared IMC powders, only with prolonged onset peak tails. In the case of q^+^ = 10 °C·min^−1^, the crystallization peaks were shifted to slightly lower (by approximately 15 °C) temperatures, and the portion of the α-IMC crystalline phase is significantly increased. This may be interpreted as the γ-phase nucleation being markedly suppressed at 10 °C (compared to the previous 25 °C case) and the acceleration of the crystal growth at higher q^+^ being driven by the growth of the α-phase from the occasional pre-existing nuclei. The last set of graphs in Figure 2 are denoted “100 days” and correspond to the DSC crystallization data for the samples that were left at laboratory temperature (~25 °C) and humidity for 100 days. For these samples, it is clear that under all conditions, the crystallization peaks are significantly smaller, flattened, and shifted to lower T. The wider but overall accelerated crystallization signals indicate a large variety of crystallization centers, which corresponds to the very long time allowed for the sub-T_g_ nucleation process. This effect is especially dominant for fine powders, where the width of the distribution of the crystallization times is increased by the vast presence of mechanical defects (acting as additional crystallization centers). The increased variety of the crystallization centers and the associated morphology of the crystallites is also indicated by the slow and gradual onset of the melting peak (as opposed to the sharp onsets in the case of the as-prepared or 14-day-old materials). There are two main reasons for such behavior, firstly because of the increased number of nuclei formed during the 100 days, and secondly, possibly because of the already developed (via GC growth) crystallites. This will be further commented upon at the end of this section, along with the Raman spectroscopy data.

**Figure 3:**
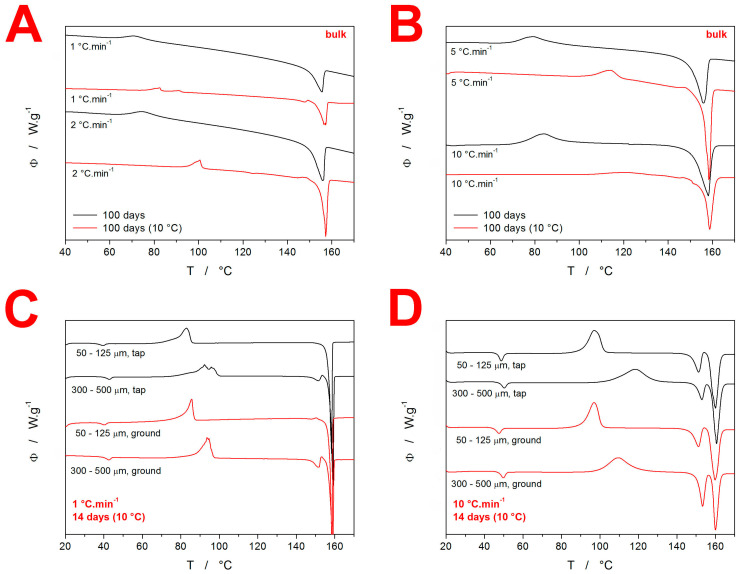
(**A**,**B**) Comparison of the DSC curves for the bulk IMC samples stored at either the laboratory temperature or at 10 °C. Exothermic signals evolve in the upwards direction. (**C**,**D**) Comparison of selected DSC curves for the IMC powders stored for 14 days at 10 °C and prepared either by gentle tapping or by forced grinding.

Additional DSC data are shown in Figure 3. The DSC curves shown in Figure 3A,B show the comparison of the “100 days” bulk data for the samples stored either openly stored in the laboratory (at ~25 °C) or desiccated at 10 °C. The bulk pieces stored at the lower temperature clearly show that the crystallization shifted to higher temperatures, with slight traces of the α-IMC phase being formed. This is in good correspondence with the difference in behaviors of the fine powders (50–125 µm) aged for 14 days, which indicates that the prolonged aging (for 100 days) follows the same qualitative pattern, and only the degree of achieved crystallinity and/or nucleation density increases to the level of the bulk samples behaving similarly to that which was previously only characteristic of the fine powders with high surface/volume ratios. The most important information is, however, that all IMC powder fractions (except for the abovementioned bulk pieces) stored at 10 °C for 100 days were fully crystalline—completely white powders, with no traces of the glass transition or crystallization effects on the DSC heating curves (the example curve is shown in the Supplemental Materials). Additional supplemental DSC data are shown in Figure 3C,D, displaying the comparison between the selected IMC particle size fractions (desiccated at 10 °C for 14 days) powdered by two distinct methods—by gentle tapping and by grinding with considerable force. Although the latter method produced a larger number of mechanical defects, as was clearly recognizable even by the naked eye, the changes in the crystallization behavior were rather negligible. The only identifiable effect was that of the slightly larger amount of the α-IMC phase being formed by the forced grinding preparation route, which is in agreement with the idea of the metastable α-phase growth being accelerated by the presence of mechanical defects.

### 3.2. Raman Spectroscopy

**Figure 4:**
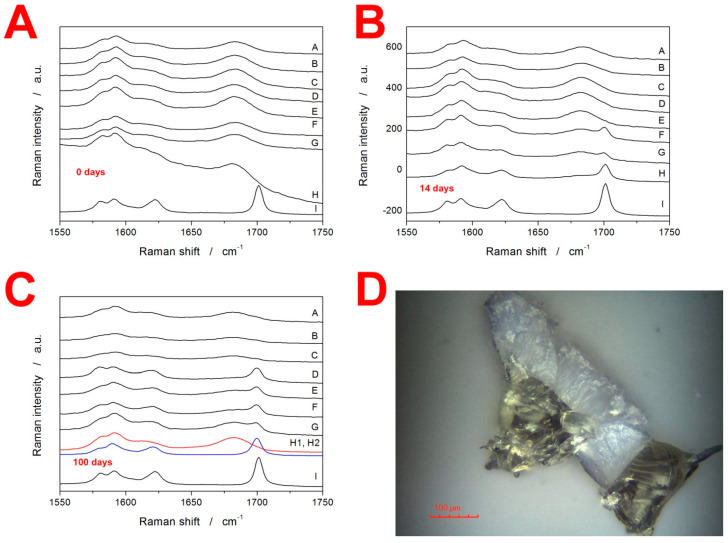
(**A**–**C**) Raman spectra of IMC powders stored for 0, 14, and 100 days at laboratory temperature and humidity. The notation of the spectra is as follows: (A) 20–50 µm, (B) 50–125 µm, (C) 125–180 µm, (D) 180–250 µm, (E) 250–300 µm, (F) 300–500 µm, (G) bulk (~500–1000 µm), (H) true bulk IMC formed as a droplet of molten IMC being allowed to cool/freeze-in on a microscopy slide, and (I) initial as-purchased IMC powder used to prepare the amorphous IMC. In graph (**C**), two spectra are displayed for sample H—one for the formed crystal and one for the free smooth surface. (**D**) Optical micrograph of a bulk sample stored for 100 days at 10 °C. The sample was then gently broken; the micrograph shows two pieces on the cross-section. The pale/white parts represent the surface crystalline layer formed by the GC growth mechanism.

Apart from the DSC, Raman spectroscopy was used to gain insight into the crystallization behavior of IMC. In particular, Raman spectroscopy is a nondestructive technique suitable for characterizing the initial/starting state of the material before the DSC measurements. Note that the DSC only implies the initial state of matter based on its thermal behavior manifesting during the consequent heating. The Raman data for the present freely stored samples at laboratory temperature and humidity are shown in Figure 4A–C, where each graph contains the following spectra: (A) 20–50 µm, (B) 50–125 µm, (C) 125–180 µm, (D) 180–250 µm, (E) 250–300 µm, (F) 300–500 µm, (G) bulk (~500–1000 µm), (H) true bulk IMC formed as a droplet of molten IMC being allowed to cool/freeze-in on a microscopy slide, and (I) initial as-purchased IMC powder used to prepare the amorphous IMC. The most important bands in the displayed Raman spectra can be assigned as follows: the amorphous IMC is characterized by the broad Raman band at 1685 cm^−1^, γ-IMC is characterized by the 1700 cm^−1^ band (benzoyl C=O stretching), and α-IMC is characterized by bands at 1650 (benzoyl C=O stretching), 1680 (benzoyl C=O stretching), and 1692 cm^−1^ (acid O–C=O stretching) [29,37].

The Raman spectra in Figure 4A correspond to the as-prepared IMC powders and confirm their amorphous character (note that spectrum I shows a comparative record for the fully crystalline γ-IMC phase). It should also be noted that due to the instrumental nature of Raman microscopy, the displayed spectra needed to be treated statistically: for each spectrum, 10–15 spots were explored on several grains randomly selected from the given sample. In cases when an amorphous spectrum is shown, all spots showed similar amorphous signals. In the case of the spectra indicating crystalline content, the spectra were always represented overwhelmingly frequently (>80% of the measurements); however, sometimes, the odd spot with a significantly higher amount of the amorphous phase could be found (especially for the coarser powders with a high d_aver_—significantly higher crystallinity than that displayed in the spectra was never found. In Figure 4B, the Raman spectra for the freely stored IMC powders aged 14 days are displayed. It is very interesting to observe that while the spectra of finely powdered IMC samples only show extremely weak traces of the γ-IMC phase, for the coarse powder fractions (300–500 µm and ~500–1000 µm), a significant portion of the material is already crystalline. Note that in the case of the true bulk IMC (sample H), the γ-IMC crystallites formed only at the spots that were purposefully scratched by a needle after the freeze-in of the droplet. 

This is more intelligibly shown in Figure 4C (aging for 100 days), where two Raman spectra are shown for the sample H—one for the formed crystallite and one for the surrounding amorphous phase. The spectra of powders aged for 100 days show that a significant amount of the crystalline content occurs for powders with a d_aver_ ≥ 215 µm (180–250 µm fraction). In the case of the finer powders, small traces of the γ-IMC crystallinity can be found, but the absolute majority of the material is still amorphous. Note that all freely stored IMC powders were still yellow after 100 days, meaning that the amorphous phase was dominant in all of them. These results are incredibly important with respect to the initiation of the GC (diffusionless glass–crystal) growth mechanism. Whereas the sub-T_g_ GC crystal growth clearly occurs in amorphous IMC and the presence of microcracks or other mechanical defects is a mandatory condition for its initiation (as demonstrated by the tests on the sample(s) H), the large concentration of these defects very effectively inhibits this type of growth, probably due to the lack of defect-free spaces through which the GC growth (morphologically manifesting either in the needle of a leaf shape) could continue. However, this finding has to also be considered from the point of view of the other set of samples stored at 10 °C, which turned fully crystalline after 100 days of storage. Two possible explanations can be derived for this difference: Firstly, the air humidity (although not detected via TGA) could slightly increase the molecular mobility on the sample surfaces of the freely stored powders, which would increase the self-diffusion tendency of IMC molecules and, consequently, cease the GC growth (this might also be an alternative explanation for the resistance to the crystal growth in the finest freely stored powders—see Figure 4A–C). A second explanation could be associated with the structural relaxation process. In [25], the structural relaxation of amorphous IMC was described in terms of the Tool–Narayanaswamy–Moynihan model [38,39,40] with the following set of phenomenological parameters: apparent activation energy ∆h^*^ = 342 kJ·mol^−1^, pre-exponential constant ln(A/s) = −127.35, the parameter of nonexponentiality β = 0.53, the parameter of nonlinearity x = 0.32. Based on this description, the theoretical simulations predict that at 25 °C, the metastable (undercooled liquid) equilibrium would be reached within 10 days, whereas at 10 °C, the material would still be far from the metastable equilibrium (fictive temperature T_f_ ≈ 15 °C) even after the 100 days. This means that at 25 °C, the glass-formation stresses within the sample would be removed relatively quickly, but would persist in the powdered material at 10 °C, which may be the cause for the accelerated (or not ceased) GC growth under the latter conditions, i.e., the positive effect of the stress-induced growth would overcome the growth-negating effect of sterical restrictions along the microcracks.

### 3.3. X-ray Diffraction Analysis and Optical Microscopy

The supplementary characterization of the IMC samples was also performed using XRD analysis—the amorphous character of the as-prepared powdered (50–125 µm) IMC was verified (see the Supplemental Materials). Furthermore, optical microscopy was used to investigate the products of the sub-T_g_ GC growth in the case of the bulk sample (~500–1000 µm) aged for 100 days in a desiccator at 10 °C. In Figure 4D, two pieces of a fractured grain are displayed on the cross-section. The pale/white color indicates the crystalline layer, and the yellowish part corresponds to the original amorphous matrix. Roughly estimated, during the 100 days, a 100 ± 5 µm thick layer formed on the surface of the particles. This translates into a growth rate of 7 × 10^−4^ µm·min^−1^ (1.2 × 10^−11^ m·s^−1^), which is in almost perfect correspondence with the value for the GC bulk growth reported in [33]. This is also very important with respect to the general crystallization behaviors manifesting during the two types of long-term storage. Whereas at 10 °C the dominant method of crystalline phase formation is consistent with the diffusionless GC growth, at 25 °C (which is still well below T_g_ of IMC), this type of growth needs to be considered as largely hindered (as opposed to the competing theory of the growth at 10 °C being accelerated). This finding might favor the increasing surface mobility concept of H_2_O molecules assisting the surface self-diffusion of IMC (suggested in the previous paragraph as one of the possible reasons for the lower degree of crystallinity reached during the long-term storage of the freely stored powder).

## 4. Discussion

### 4.1. Quantification of Thermal Behavior

**Figure 5:**
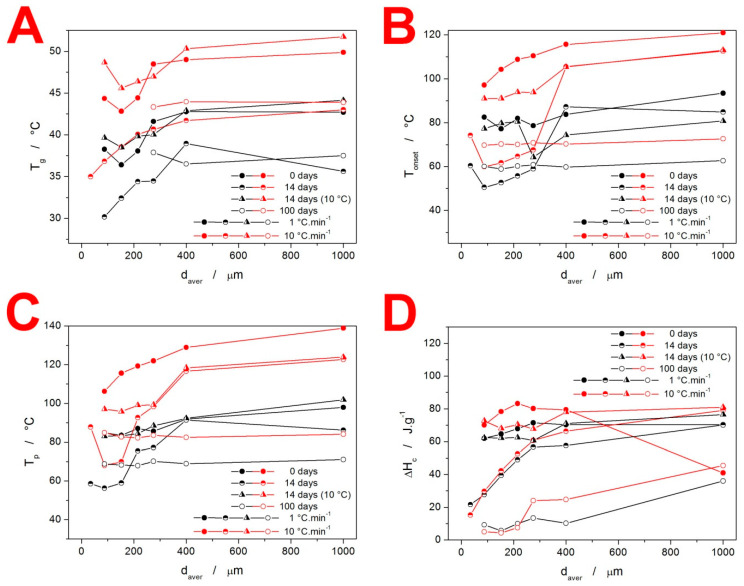
Base characteristic quantities (glass transition temperature T_g_—(**A**), the onset temperature of the crystallization peak T_onset_—(**B**), the peak temperature of the crystallization peak T_p_—(**C**), and crystallization enthalpy ΔH_c_—(**D**) obtained at chosen q^+^ for the IMC powders stored under different conditions.

In the first part of this section, the quantification of the thermal behavior of the IMC powders will be introduced. In particular, the characteristic temperatures and enthalpy changes associated with the glass transition, crystallization, and melting phenomena will be reported. The evolution of T_g_ with d_aver_, q^+^, and storage type is shown in Figure 5A. The most pronounced feature displayed in these dependencies is the significantly lower (by approximately 7 °C) T_g_ values recorded for the freely stored samples stored for 14 and 100 days. Considering that the TGA results did not detect a significant amount of adsorbed water (that would be released at T < 100 °C), the hydroplasticization effect can probably be ruled out as a reason for the decreased T. It should also be noted that whereas a significant lowering of T_g_ by a very small water content can be expected for polymers, where just a few water molecules impregnated between the polymeric chains can spread them apart, this concept is not valid for small organic molecules such as IMC where a large amount of the H_2_O molecules (certainly detectable by TGA) would have to be absorbed by the amorphous material for its structure to become diluted and more mobile in bulk. Similarly, the potential presence of a larger number of nuclei also cannot in any way explain the lowered T_g_—the presence of a crystalline phase would make the overall matrix more rigid (increasing T_g_), and the nucleation proceeds primarily at the surface of the amorphous grain, which would not significantly influence the bulk mobility/self-diffusion. This leaves, as the most probable hypothesis, the association with the difference in the structural relaxation behavior. The release of the quench-in stress may lead to a different ordering of the amorphous structure (akin to the difference between the α and γ polymorphs), which might explain the slightly decreased T_g_ in the case of the materials stored at 25 °C. For the powders stored at 10 °C, the stress introduced into the structure during the glass formation is not removed, and the pseudo-equilibrium is not achieved—hence similar glass transition kinetics to that of the as-prepared (quenched-in) IMC.

The evolution of characteristic temperatures (extrapolated onset T_ons_ and peak maximum T_p_) and enthalpies of the crystallization process for the present IMC samples are shown in Figure 5B–D. As is apparent from the comparison of the two characteristic temperatures, they exhibit very similar courses for the dependencies. Further, the freely stored IMC powders tend to exhibit lower T_ons_ and T_p_ values by 20–30 °C. In correspondence with the comments of Figure 2, the characteristic temperatures show the markedly higher effect of the γ-phase nucleation on the acceleration of the overall crystallization process in the case of the IMC powders stored at 25 °C. Whereas the storage at 10 °C also prolongs the low-T tail of the DSC crystallization peaks (see Figure 2), the maximum rates of the amorphous-to-crystalline transformation are still close to those of the original as-prepared material.

Very important is Figure 5D, where the most noteworthy data are the very low ΔH_c_ obtained for low q^+^ and freely stored IMC powder samples. Since the melting enthalpies did not drastically change (as will be discussed below), a similar degree of crystallinity was achieved within the combination of the GC growth and above-T_g_ thermally induced growth. However, as was shown in Figure 4, the GC growth is practically nonexistent in the case of the freely stored powders with d_aver_ < 275 µm during the first 14 days. Hence, the crystallization process proceeding under these conditions has to be associated with a significantly lower ΔH_c_. The main lead lies in the crystallization peak occurring at a largely decreased temperature (by over 20 °C, see Figure 2 and Figure 5C). This has two consequences: Firstly, the naturally decreased ΔH based on Kirchhoff’s law certainly plays a nonnegligible role. Secondly, the nucleation at 25 °C is rapid and the γ-IMC crystal growth originating primarily from a large number of nuclei at mechanically induced defects (as evidenced by the low-T shoulder of the crystallization peak becoming dominant in Figure 2) may result in the formation of a morphologically different phase, associated with a significantly lower evolved heat. 

**Figure 6:**
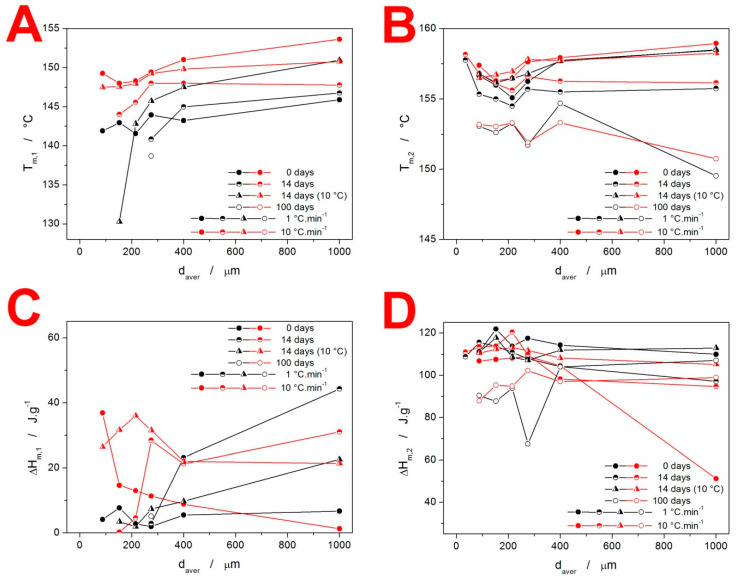
Graphs showing T_m,1,_ (**A**) T_m,2,_ (**B**) and the corresponding melting enthalpies ΔH_m,1_ (**C**) and ΔH_m,2_ (**D**) obtained at chosen q^+^ for the IMC powders stored under different conditions.

The melting behavior of the present IMC samples is quantified in Figure 6. The T_m_ temperatures (indexed “1” for the α-IMC phase melting peak and “2” for the γ-IMC phase melting peak) demonstrate the reproducibility and variability in their determination when an increasingly larger variety of the size/morphology of the forming crystallites occurs. The most important data are depicted in Figure 6C,D. The values of ΔH_m1_ clearly indicate the conditions for the formation of the α-IMC phase, as already discussed in Figure 2. For the as-prepared IMC, while it is the typical combination of low d_aver_ and high q^+^, the 14-day-stored samples show a markedly higher tendency toward the formation of the α-phase—increased content of these crystallites occurs for all coarse powders, and even for fine powders stored at 10 °C and heated at high q^+^. The ΔH_m2_ data shown in Figure 5D indicate that under practically all circumstances, the quality of the formed crystalline phase and the achieved degree of crystallinity are similar. The only exception is for the IMC powders stored for 100 days, where the below-T_g_-formed GC crystallites may have a lower density filled-in crystalline phase and thus an overall lower ΔH_m_. Note that the outlying point with a d_aver_ = 1000 µm in the dependence for the as-prepared samples is caused by the interference between the exothermic crystallization and endothermic melting DSC peaks at high q^+^.

### 4.2. Crystallization Kinetics

**Figure 7:**
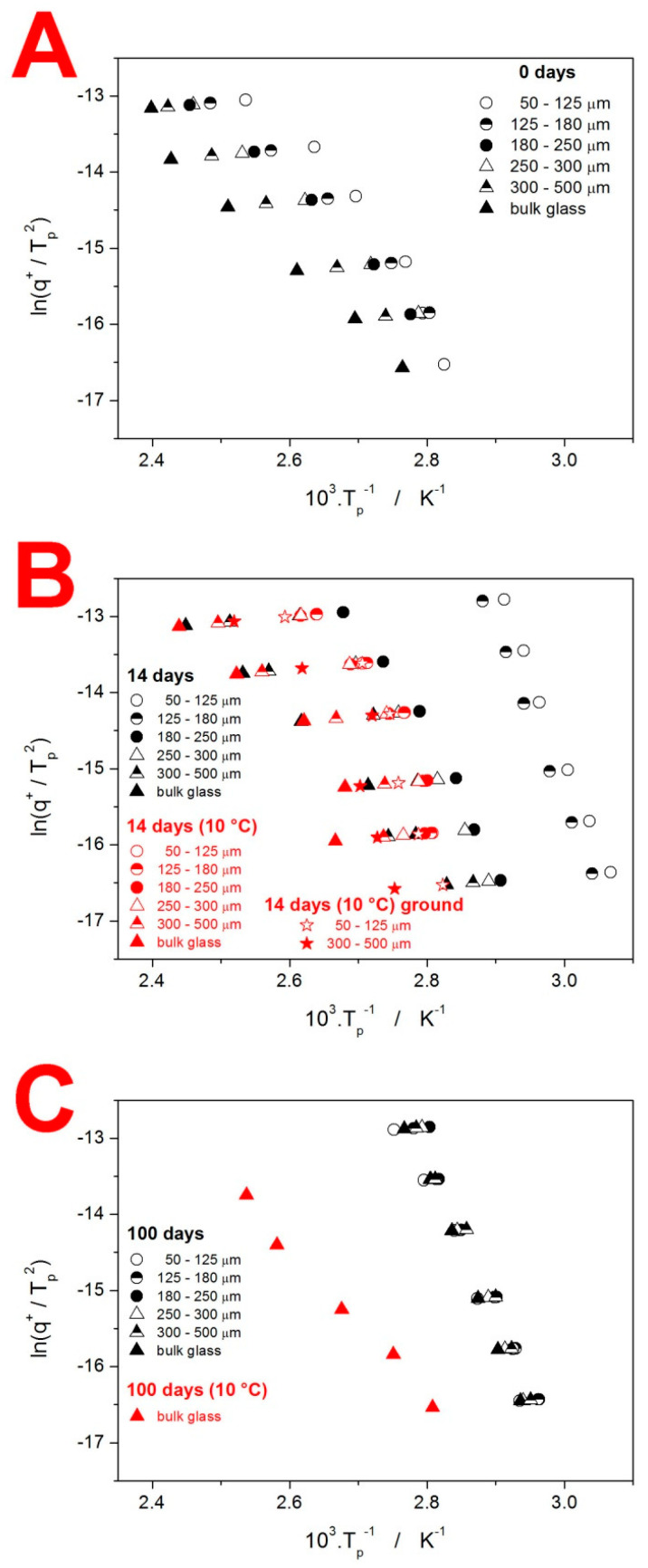
Kissinger plots for the IMC powders stored under different conditions. Each graph displays data for one time period: 0 days = as-prepared (**A**), 14 days (**B**), and 100 days (**C**).

The crystallization kinetics are commonly quantified based on the standard DSC kinetic Equation (1) [41]:

(1) $$f\left(\alpha \right)=\alpha^{M_{AC}}{\left(1-\alpha \right)}^{N_{AC}}$$

where Φ is the measured heat flow, Δ*H* is the crystallization enthalpy, A is the pre-exponential factor, *E_c_* is the activation energy of the macroscopic crystallization, *R* is the universal gas constant, *T* is temperature, and *f*(*α*) is an expression for a kinetic model with α standing for the degree of conversion from the amorphous to the crystalline state. The determination of the activation energy E can be very conveniently derived using the Kissinger Equation (2):

(2) $$\ln\left(\frac{q^{+}}{T_{p}^{2}}\right)=-\frac{E_{c}}{RT_{p}}+const.$$

where *T_p_* is the temperature corresponding to the maximum of the crystallization peak. The so-called Kissinger plots (dependencies based on Equation (2)) are for the IMC powders shown in Figure 7—note the similar scaling in all three graphs. 

The crystallization of the as-prepared IMC powders (see Figure 7A) exhibits typical features, i.e., the increase in T_p_ with q^+^ and d_aver_. Worth noting is also the curvature of the dependencies obtained for the fine IMC powders. This deviation from the linear course, expected for a single uniform crystallization process, is caused by the change in the crystal growth mechanism, as evidenced by the shift in the asymmetry of the peak (see the differences in peak shape between the data for q^+^ = 1 °C·min^−1^ and q^+^ = 10 °C·min^−1^ in Figure 2). The data for the powders stored for 14 days are depicted in Figure 7B. Whereas the samples stored at 10 °C do not exhibit major shifts in comparison to the as-prepared IMC, the data for the powders stored at the laboratory temperature spread to a significantly lower T, which is the consequence of the nucleation proceeding (primarily) on the mechanically induced defects, accelerating the overall macroscopic crystallization. Interestingly, at 100 days of storage at laboratory conditions, the crystallization process becomes markedly unified due to the nucleation saturation, and, importantly, due to the crystalline phase being formed (via GC growth mechanism; see Figure 4C) at the energetically most favorable sites (which cancels the crystallization acceleration apparent in Figure 7B for the fine powders).

**Figure 8:**
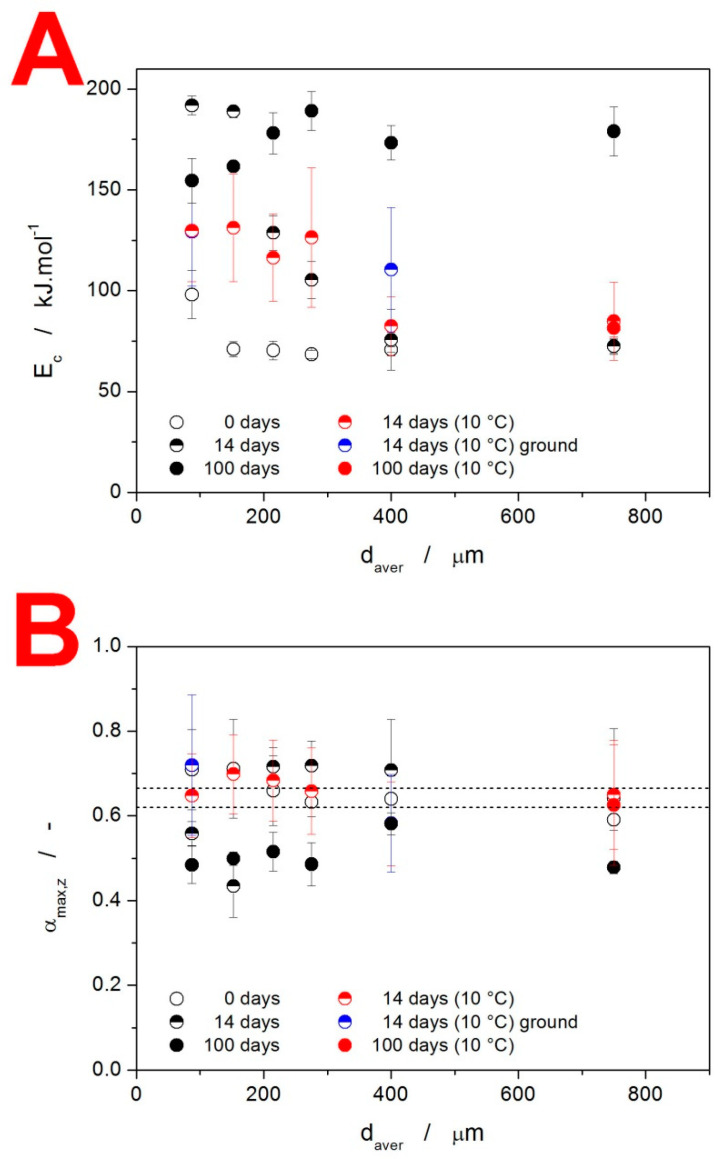
(**A**) Activation energy of the crystallization process E_c_ determined using Equation (2) from the Kissinger dependencies displayed in Figure 7. (**B**) Values of the degree of conversion corresponding to the maxima of the characteristic kinetic functions z(α), see Equation (3), determined for the IMC powders stored under various conditions. The dashed horizontal lines indicate the applicability range of the JMA kinetic model.

The base quantification of the crystallization mechanisms manifesting in the as-prepared and stored IMC powders is introduced in Figure 8. The apparent activation energies E_c_ determined from the Kissinger dependencies (see Figure 7) are shown in Figure 8A. A clear increase in E_c_ with d_aver_ as well as with the time of storage needs to be confronted with the overall decrease in the crystallization temperature, for which the pre-exponential factor A (see Equation (1)) is responsible. As such, the process should not be mechanistically interpreted in accordance with the general Arrhenian concept, i.e., that the energetic barriers represented by E increase due to, e.g., sterical restrictions, and the incidence of molecules from the amorphous phase being attached to the crystalline frontline is increased via A due to significantly more nucleated samples or simply a larger amount of potential crystal growth sites being present. Instead, we should consider the E values as truly apparent, primarily driven by the relative facilitation of the crystallization process—as presented, e.g., in Figure 2 and Figure 7. In this regard, the storage for 14 days results in particularly accelerated growth at higher q^+^ (probably as a consequence of the mixed-in formation of α-phase with a significantly higher growth rate [25,28,29]). On the other hand, the storage for 100 days led to a large amount of γ-phase formation, already at the laboratory temperature (via the GC growth mechanism), which further accelerated the crystal growth in coarse IMC powders—hence the uniformity of the crystallization temperatures, as evidenced in Figure 7C.

The asymmetry of the crystallization peaks is described in terms of the characteristic kinetic function *z*(*α*) [42]

(3) $$z\left(\alpha \right)=\Phi \cdot T^{2}$$

where the degree of conversion corresponding to the maximum of this function is denoted *α_max,z_*. For certain kinetic models, this quantity exhibits characteristic values. This is particularly relevant for the nucleation–growth Johnson–Mehl–Avrami (JMA) model [43,44,45,46] (Equation (4)) with the kinetic exponent *n_JMA_*, for which *α_max,z_* = 0.632. The statistical limits for this value were recently [42] shown to be 0.620–0.665 for the correlation coefficient r^2^ = 0.999.

(4) $$f\left(\alpha \right)=n_{JMA}\left(1-\alpha \right){\left[-\ln\left(1-\alpha \right)\right]}^{1-\left({1/n}_{JMA}\right)}$$

The *α_max,z_* values for the present samples are shown in Figure 8B, together with the abovementioned theoretical limits for the applicability of the JMA model. For each displayed point, the averaging was carried out for all applied q^+^. The large error bars indicate the trends in the asymmetry of the peaks with *q*^+^. As a consequence, only a small fraction of the measured data actually falls within the limits of JMA-model applicability. Instead, the more flexible alternative in the form of the semiempirical autocatalytic Šesták–Berggren (AC) model [41] (Equation (5)) with kinetic exponents *M_AC_* and *N_AC_* will have to be used.

(5) $$f\left(\alpha \right)=\alpha^{M_{AC}}{\left(1-\alpha \right)}^{N_{AC}}$$

### 4.3. Kinetic Predictions

**Figure 9:**
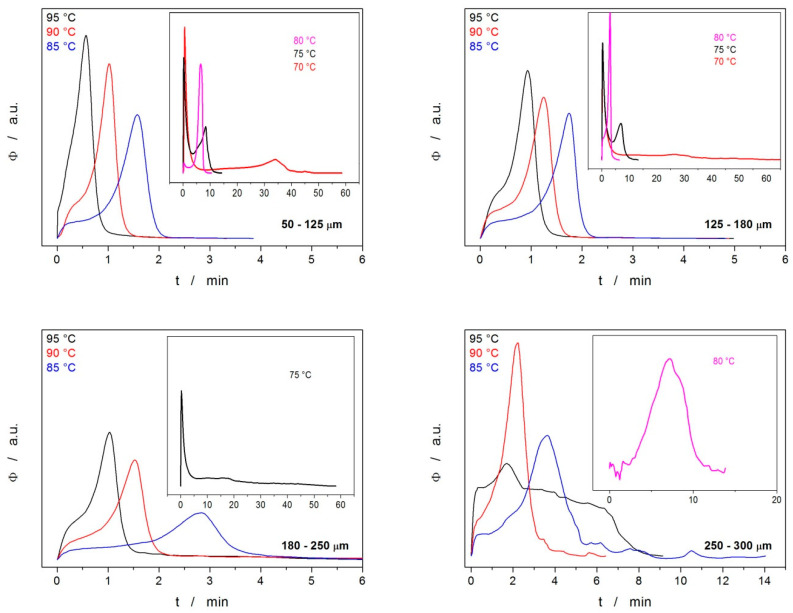
DSC curves for the isothermal crystallization experiments that exhibited detectable and reproducible signals—generally, fine powders annealed at higher temperatures. Exothermic signals evolve in the upwards direction.

Since the main goal of the kinetic analysis was to provide reasonable predictions for extrapolated measurement conditions, a series of isothermal measurements were performed at different annealing temperatures T_a_ for the powdered IMC samples. The DSC data are shown in Figure 9, where the insets display the curves obtained at lower T_a_s, for which significantly higher annealing times were needed.

It is immediately apparent that at low T_a_s, the crystallization process is complex, consisting of two peaks, where one of the processes proceeds very quickly and most probably corresponds to the growth on the energetically preferential sites (mechanical defects), whereas the second process is slow and should correspond to the normal growth from the sample surface. At higher T_a_s, both processes merge, and, interestingly, the normal growth dominates (here, T is probably high enough for the normal growth to proceed preferentially throughout the whole sample). The threshold for the separation of these two cases appears to be at ~80 °C, with a slight dependence on d_aver_. Note the resemblance with similar separation in the case of the nonisothermal data obtained for the fine powders stored for 14 days at the laboratory temperature (see Figure 2). The data in Figure 9 also show that for coarser powders, the crystallization behavior becomes less reproducible, exhibiting a higher dependence on the quality of the surface of the few particular IMC grains included in the given sample. The isothermal data have become unsatisfactorily irreproducible for the particle size fractions ≥ 300–500 μm. 

The isothermal DSC data were used as an experimental reference for the predictions made based on the kinetic description of the nonisothermal data via the combination of Equations (1) and (5). The actual enumeration of these equations was based on the single-curve multivariate kinetic analysis sc-MKA [47]:

(6) $$RSS=\sum_{j=1}^{n}\sum_{k=First_{j}i}^{Last_{j}}w_{j,k}{\left(Y\exp_{j,k}-Ycal_{j,k}\right)}^{2}$$

(7) $$w_{j}=\frac{1}{{\left|{\left[d\alpha /dt\right]}_{\max}\right|}_{j}+{\left|{\left[d\alpha /dt\right]}_{\min}\right|}_{j}}$$

where *RSS* is the sum of the squared residue, *n* is the number of measurements, *j* is the index of the given measurement, *First_j_* is the index of the first point of the given curve, *Last_j_* is the index of the last point of the given curve, *Yexp_j,k_* is the experimental value of the point *k* of curve *j*, *Ycal_j,k_* is the calculated value of the point *k* of curve *j*, and *w_j_* is weighting factor for curve *j*. The sc-MKA method applies a fixed E_c_ (in the present case at values from Figure 8A) to each DSC curve individually so that trends in the kinetic parameters can be obtained. 

The tests of the predictive ability of this description were performed for four cases: isothermal annealing of the 50–125 µm powder at 70 and 90 °C, and isothermal annealing of the 250–300 µm powder at 80 and 90 °C. For each of these cases, the predictions were made based on the kinetic parameters describing the nonisothermal DSC data obtained either at 0.5 or at 20 °C·min^−1^ (the data are listed in Table 1).

**Figure 10:**
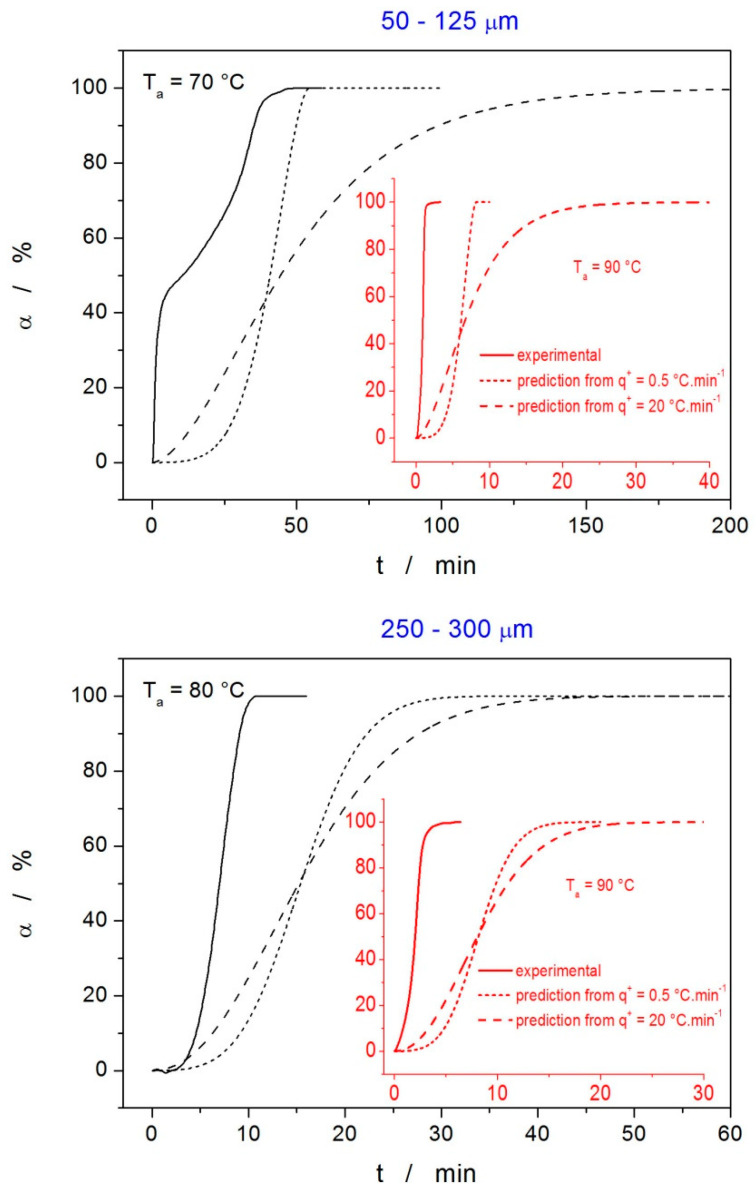
Tests of the kinetic prediction accuracy for isothermal crystallization based on the description of nonisothermal DSC data—the kinetic parameters of this description are listed in Table 1. For each of the two tested powders (50–125 µm and 250–300 µm), two annealing temperatures were selected and the corresponding isothermal crystallization experiments performed—solid lines represent the α–t data. For each of these four isothermal experiments, two theoretical predictions were made using the kinetic parameters obtained from the nonisothermal crystallization data measured at 0.5 and 20 °C·min^−1^.

The comparison of the isothermal experimental data and the corresponding predictions calculated from the description of the nonisothermal data are shown in Figure 10. As is apparent, the predictions calculated based on the description of the low-q^+^ nonisothermal data are closer to the actual crystallization behavior—this was expected since the predictions were made for the extrapolation to lower T_a_s, to which the data obtained at 0.5 °C·min^−1^ are closer. However, although the extrapolation was only small, the predictions in all cases were significantly inaccurate, providing only a rough estimate for the crystallization time, precise only to several multiples of the correct value. Since this is still sufficient for pharmaceutical practice, such inaccuracy indicates the vast complexity of the indomethacin crystallization behavior, where nucleation and crystal growth of two polymorphs compete (within the sample processing timescale) with the GC growth even at the laboratory temperature. It is also noteworthy that similar predictions made for T_a_ = 25 °C suggest the full formation of the crystalline phase within 2–6 days; for T_a_ = 10 °C, the crystallization times were 6–50 days. Whereas the latter can be considered to be an acceptable rough estimate, the former completely false prediction further stresses the complex crystal growth behavior at temperatures around T_g_.

## 5. Conclusions

An extensive exploration of the crystallization behavior of amorphous indomethacin was performed by means of nonisothermal DSC experiments. The measurements were particularly focused on the influences of particle size (powdered samples), heating rate, and storage conditions. The main experimental findings can be summarized as follows:

- The preferential formation/dominance of particular polymorphs is consistent and well reproducible, with the increased formation of the α-IMC phase being associated with high T (and mobility in general), the presence of mechanical defects, and long-term nucleation at the free surface.
- Whereas the nonisothermal crystallization proceeds uniformly (in an apparent single process), isothermal DSC crystallization data show that below ~80 °C, the significantly faster crystal growth from the mechanical defects proceeds independently from the growth at the free surface of the IMC grains.
- At 10 °C and zero humidity, the GC growth proceeds during long-term storage in accordance with the literature reports on crystal growth rate (~100 µm of the γ-IMC phase in 100 days).
- At 25 °C (still well below T_g_) and laboratory humidity, the freely stored fine powder samples (with d_aver_ ≤ 250–300 µm) exhibited practically no traces of the crystalline phase during the first 14 days of storage; after 100 days, the same was still true for powders with d_aver_ ≤ 125–180 µm. This indicates marked suppression of the GC growth by the presence of mechanical defects at these conditions. This finding may open a revolutionary route to the long-term storage of amorphous APIs.
- The freely stored bulk material with no mechanical damage and a smooth surface exhibited zero traces of GC growth (as confirmed by microscopy) after >150 days of storage.

The tests on the predictive ability of the kinetic models based on the nonisothermal DSC measurements have shown that the accuracy of such theoretical simulations is rather poor, sufficient only for a rough determination of short-term processing conditions for amorphous IMC. Even in the relatively common situation, when the API has only two dominant competing polymorphs and the GC growth is negated (the comparison with the as-prepared powders), the variable particle size (associated with the presence of mechanical defects) and the difference in temperature dependencies of the nucleation and crystal growth processes contributing to the two polymorphs make the kinetic predictions very difficult. Exponentially increased complications for such predictions occur when the effects of GC growth and/or humidity are involved. For this reason, the kinetic studies of amorphous APIs should probably always include not only isothermal but also nonisothermal data in the relevant ranges as well as a verification of the correspondence between the two types of measurements. This statement may, however, not be general—possible simplifications may be implicated in the cases of APIs with a T_g_ well above (>50 °C) the storage temperature [23,24] and nonisothermal measurements performed at extremely low (<0.2 °C·min^−1^) heating rates.

## Figures and Tables

**Table 1:** Kinetic parameters determined by the sc-MKA method from the selected nonisothermal DSC data obtained for given values of d_aver_ and q^+^.

d_aver_/µm	50–125		250–300	
q^+^/°C·min^−1^	0.5	20	0.5	20
E_c_/kJ·mol^−1^	98	98	68	68
log(A/s)	12.1551	11.6612	7.6702	7.3840
M_AC_	0.8235	0.4249	0.7781	0.5538
N_AC_	0.4209	0.9941	0.8679	0.8719

## Data Availability

The original data are available upon request from the corresponding author.

## Supplementary Materials

The following supporting information can be downloaded at: https://www.mdpi.com/article/10.3390/molecules28041568/s1, Figure S1: Thermogravimetric data for the IMC samples stored for 100 days.; Figure S2: DSC curves for the IMC samples stored for 100 days. Figure S3: XRD pattern for the as-prepared IMC sample.
