# Supplementary material for: Indomethacin: Effect of Diffusionless Crystal Growth on Thermal Stability during Long-Term Storage
Source: Molecules. 2023 Feb 6;28(4):1568. doi: 10.3390/molecules28041568 (PMC9963031; doi:10.3390/molecules28041568)
Supplement: Supplementary file 1 [file molecules-28-01568-s001.zip › molecules-2201902-supplementary.pdf]

molecules
MDPI

Supplemental online material

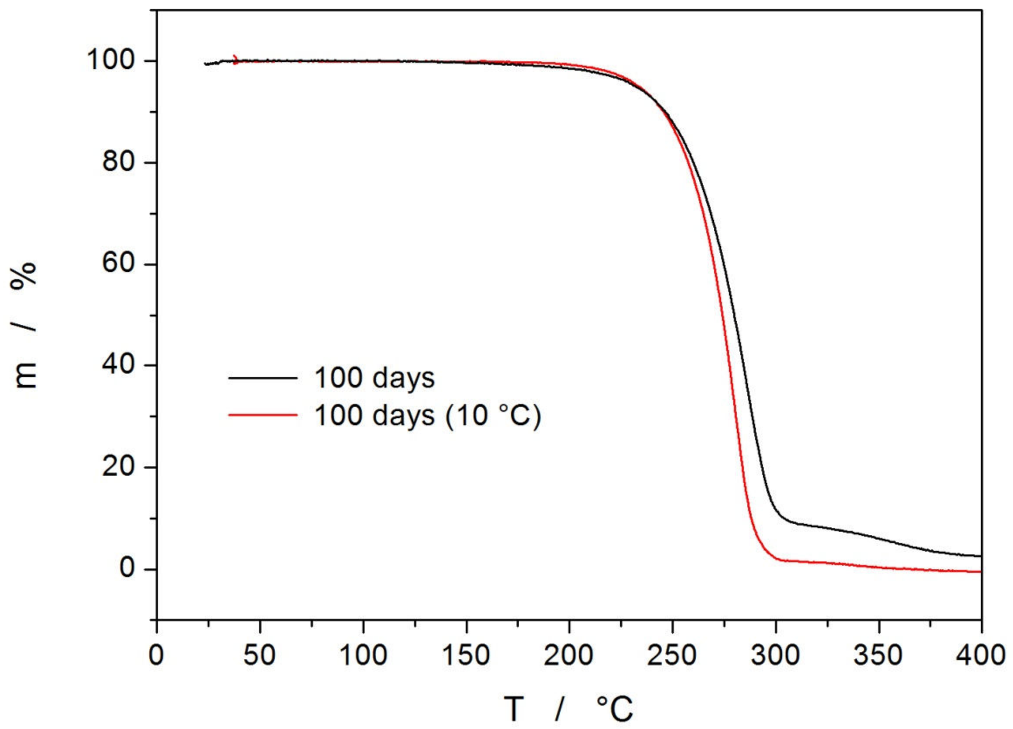

Figure S1. Thermogravimetric data for the IMC samples stored for 100 days.

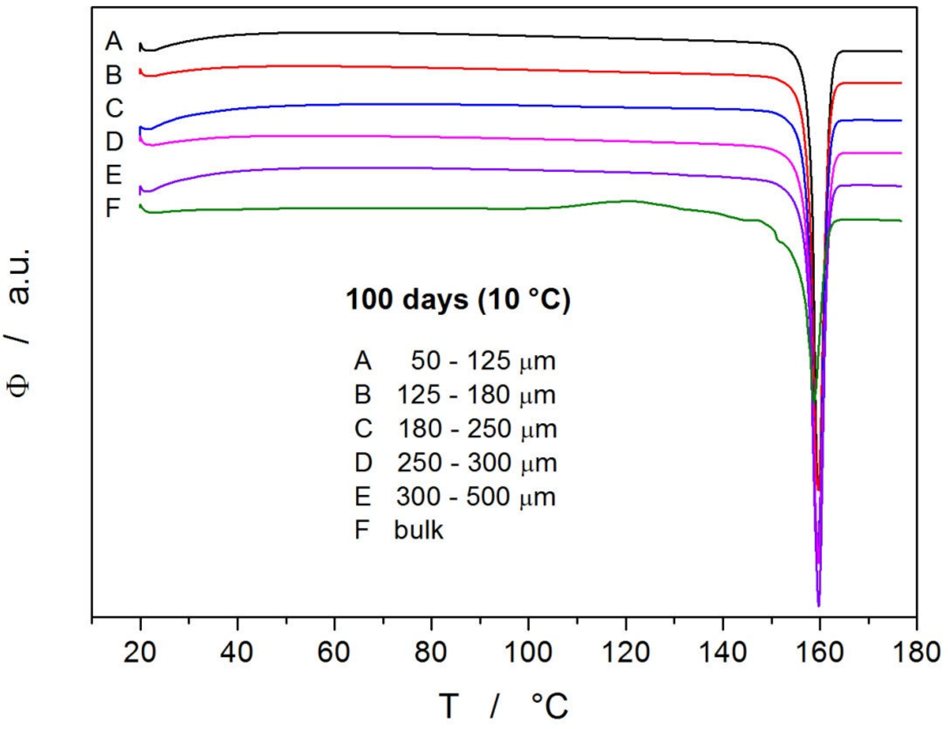

Figure S2. DSC curves for the IMC samples stored for 100 days at 10 °C.

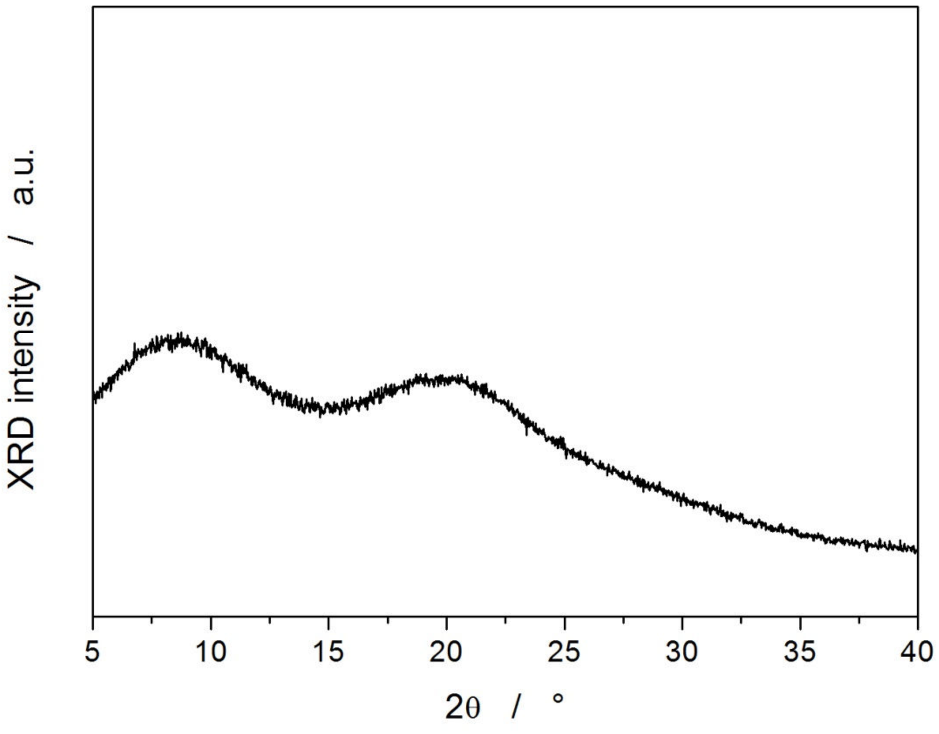

Figure S3. XRD pattern for the as-prepared IMC sample.
